# Microbial players and processes involved in phytoplankton bloom utilization in the water column of a fast‐flowing, river‐dominated estuary

**DOI:** 10.1002/mbo3.467

**Published:** 2017-03-20

**Authors:** Maria W. Smith, Lydie Herfort, Caroline S. Fortunato, Byron C. Crump, Holly M. Simon

**Affiliations:** ^1^ Center for Coastal Margin Observation & Prediction Oregon Health & Science University Portland OR USA; ^2^ Institute of Environmental Health Oregon Health & Science University Portland OR USA; ^3^ Josephine Bay Paul Center Marine Biological Laboratory Woods Hole MA USA; ^4^ Oregon State University College of Earth, Ocean and Atmospheric Sciences Corvallis OR USA

**Keywords:** Bacteroidetes, metagenome analysis, phytoplankton bloom degradation

## Abstract

Fueled by seasonal phytoplankton blooms, the Columbia River estuary is a natural bioreactor for organic matter transformations. Prior metagenome analyses indicated high abundances of diverse Bacteroidetes taxa in estuarine samples containing phytoplankton. To examine the hypothesis that Bacteroidetes taxa have important roles in phytoplankton turnover, we further analyzed metagenomes from water collected along a salinity gradient at 0, 5, 15, 25, and 33 PSU during bloom events. Size fractions were obtained by using a 3‐μm prefilter and 0.2‐μm collection filter. Although this approach targeted bacteria by removing comparatively large eukaryotic cells, the metagenome from the ES‐5 sample (5 PSU) nevertheless contained an abundance of diatom DNA. Biogeochemical measurements and prior studies indicated that this finding resulted from the leakage of cellular material due to freshwater diatom lysis at low salinity. Relative to the other metagenomes, the bacterial fraction of ES‐5 was dramatically depleted of genes annotated as Bacteroidetes and lysogenic bacteriophages, but was overrepresented in DNA of protists and Myxococcales bacterivores. We suggest the following equally plausible scenarios for the microbial response to phytoplankton lysis: (1) Bacteroidetes depletion in the free‐living fraction may at least in part be caused by their attachment to fluvial diatoms as the latter are lysed upon contact with low‐salinity estuarine waters; (2) diatom particle colonization is likely followed by rapid bacterial growth and lytic phage infection, resulting in depletion of lysogenic bacteriophages and host bacteria; and (3) the subsequent availability of labile organic matter attracted both grazers and predators to feed in this estuarine biogeochemical “hotspot,” which may have additionally depleted Bacteroidetes populations. These results represent the first detailed molecular analysis of the microbial response to phytoplankton lysis at the freshwater–brackish water interface in the fast‐flowing Columbia River estuary.

## 1 Introduction

Estuarine microbiota modulate the influence of rivers in coastal regions by remineralizing river‐borne organic matter and releasing inorganic nutrients (Jickells, 1998; Maher & Eyre, 2012; Satinsky et al., 2014), some of which are discharged by the river plume to the coastal ocean. As the second largest river in the United States in terms of water discharge volume (Sullivan, Prahl, Small, & Covert, 2001), the Columbia River's impact on the coastal Pacific Ocean is extensive (Hickey & Banas, 2003). The river‐dominated Columbia River estuary is, in return, affected by strong semi‐diurnal ocean tides (Jay, 1984). Previous studies of the estuarine microbiota showed that temporally variable tidal forcing and river discharge are major determinants of microbial community composition through formation of steep gradients of environmental factors, including salinity, temperature, oxygen concentrations, and so forth (Breckenridge, Bollens, Rollwagen‐Bollens, & Roegner, 2014; Fortunato, Herfort, Zuber, Baptista, & Crump, 2012; Fortunato et al., 2013; Herfort, Peterson, McCue, & Zuber, 2011; Smith, Zeigler Allen, Allen, Herfort, & Simon, 2013; Smith et al., 2010). Typically, the river flow peaks during the spring freshet (May–June) due to the melting of snowpack, and then decreases to low levels in late summer/early fall (Chawla, Jay, Baptista, Wilkin, & Seaton, 2008). The composition of the estuarine microbial community reflects these changing conditions through shifts in the dominant taxa (Fortunato et al., 2013).

The composition of bacterial communities in the estuary is also influenced by the sources of dissolved and particulate organic matter (DOM and POM, respectively; Fortunato et al., 2013; Herfort, et al., 2011; Simon, Smith, & Herfort, 2014; Smith et al., 2013). Fast‐flushing times (0.5–5 days; Neal, 1972) and light‐limiting turbidity results in low in situ primary production (Frey, Lara‐Lara, & Small, 1984; Lara‐Lara, Frey, & Small, 1990), and therefore the main source of POM in the estuary from spring to fall is transient allochthonous algal blooms (Frey et al., 1984; Sullivan et al., 2001). The largest diatom blooms in the Columbia River typically occur just before the spring freshet, with several smaller blooms taking place during the summer (Maier, 2014). These freshwater diatom cells lyse in the lower estuary at the freshwater–brackish water interface (salinities of 3–5 PSU; Haertel, Osterberg, Curl, & Park, 1969; Lara‐Lara et al., 1990), and are the major source of labile organic matter in the estuary (Frey et al., 1984; Prahl, Small, & Eversmeyer, 1997). Phytoplankton also bloom in the coastal ocean, and provide a smaller but significant input of organic matter to the estuary, particularly in summer and early fall (Amspoker & McIntire, 1986; Haertel et al., 1969; Herfort, et al., 2011; Roegner, Seaton, & Baptista, 2011). These coastal blooms develop in response to seasonal upwelling (Kudela et al., 2005) and are transported into the estuary during periods of low river discharge (Roegner et al., 2011; Smith et al., 2015). In August–October, estuarine blooms of the mixotrophic ciliate, *Mesodinium* spp., constitute the only autochthonous POM source from phytoplankton (Herfort, Peterson, McCue, & Crump, et al., 2011). Dead and degrading phytoplankton blooms are utilized by heterotrophic bacteria and transformed into detritus, DOM and POM that may be utilized in the estuary (Gilbert, Needoba, Koch, Barnard, & Baptista, 2013), transported to the coastal ocean with the river plume (Small et al., 1990), or deposited and utilized in estuarine sediments (Smith et al., 2015).

In contrast to most North American estuaries, the fast‐flushing Columbia River estuary is commonly considered a detritus‐based ecosystem (Simenstad, Small, & McIntire, 1990; Small et al., 1990). The detritus consumption processes in the estuary are determined by physical forcing through water mixing and sediment accretion and erosion (Simenstad et al., 1990). Because of the low water residence times that are typical of this estuary (Neal, 1972), most of the heterotrophic remineralization is thought to occur on particles, especially in retentive areas that increase particle residence time, such as estuarine turbidity maxima (ETM; Crump & Baross, 1996; Crump, Baross, & Simenstad, 1998). Therefore, much work has been focused on Columbia River ETM, and particularly on characterization of microbial composition and activities therein (Crump & Baross, 1996; Crump et al., 1998; Herfort, et al., 2011; Simenstad, Morgan, Cordell, & Baross, 1994; Smith et al., 2013). However, based on microscopic and biogeochemical data, substantial conversion of phytoplankton carbon to detrital carbon has been shown to take place at the freshwater–brackish water interface (Small et al., 1990), suggesting that ecologically important transformations of organic matter also occur outside of previously identified particle‐retention areas. In fact, recently developed numerical simulations of water mass transport (Karna & Baptista, 2016; Karna et al., 2015) indicated pronounced water retention in the lower Columbia River estuary. Despite generally shorter water retention times during the periods of low river flow (August–October), the calculated water age can reach >50 hr in the channels, with the oldest age observed for mixed waters masses of intermediate salinities (T. Karna, personal communication). Such extended water residence provides an opportunity for organic matter remineralization in the water column mediated by estuarine microbiota, but earlier studies on bacterial processes in the Columbia River estuary have generally overlooked these potential “hotspots” of activity.

Our prior results indicated that deposition of marine diatom particles in the sediments of a brackish lateral bay (Youngs Bay) in the Columbia River estuary was associated with a dramatic enrichment of Bacteroidetes taxa (~60% of total Bacteria) and corresponding genes involved in phytoplankton degradation (Smith et al., 2015). Members of this taxon are commonly found to dominate phytoplankton bloom‐associated bacterial communities (Buchan, Lecleir, Gulvik, & Gonzalez, 2014), and more specifically are involved in the initial degradation of coastal blooms (Teeling et al., 2012). Global analysis of metagenomes and metatranscriptomes from estuarine water samples also indicated that multiple members of the Bacteroidetes group were observed at highly variable water salinities throughout the euphotic zone, altogether constituting between 20% and 40% of all water column bacteria (Fortunato & Crump, 2015; Smith et al., 2013). Our focus in this report was on assessing the involvement of Bacteroidetes taxa in mechanisms of POM turnover in the Columbia River estuary (with potential relevance to other fast‐flowing estuaries). To accomplish this goal, we reanalyzed metagenome data from the 0.2 to 3 μm fractions of five water samples collected across a salinity gradient (0, 5, 15, 25, and 33 PSU salinities in the river, estuary, and plume) during active phytoplankton bloom formation. The original study was performed to analyze the response of free‐living bacteria to environmental gradients in the estuarine water column, thus the prefilter fractions were not retained. The general properties of the metagenome sequences are described in Fortunato and Crump (2015), indicating the presence of taxonomically variable bacterial communities along the salinity gradient in the riverine, estuarine, and oceanic water masses. Analysis of environmental metadata revealed that all five whole water samples contained moderate amounts of phytoplankton. However, only one of the five analyzed free‐living fractions, namely ES‐5 (from the 5 PSU estuarine water sample), contained tens of thousands of diatom sequences. Since live diatom cells could not pass through the 3‐μm prefilter (and indeed they did not in four of the five samples), the sequences present in the smaller size fraction indicated diatom cell death. Here, we report results of more detailed analyses on phytoplankton utilization by the heterotrophic estuarine community. Our data indicate that Bacteroidetes taxa are indeed major players in the initial response of heterotrophic bacteria to phytoplankton cell lysis at the estuarine freshwater–brackish water interface. Our results suggest that Bacteroidetes taxa are recruited to diatom detritus as cells lyse in low‐salinity estuarine waters, playing key roles in the initial turnover of cellular debris. Comparative metagenomic analysis was also performed on both estuarine water and sediment samples (Smith et al., 2015), indicating distinct Bacteroidetes taxa are involved in organic matter degradation in the water column vs. sediments.

## 2 Materials and Methods

### 2.1 Sample collection and DNA isolation

**Figure 1:**
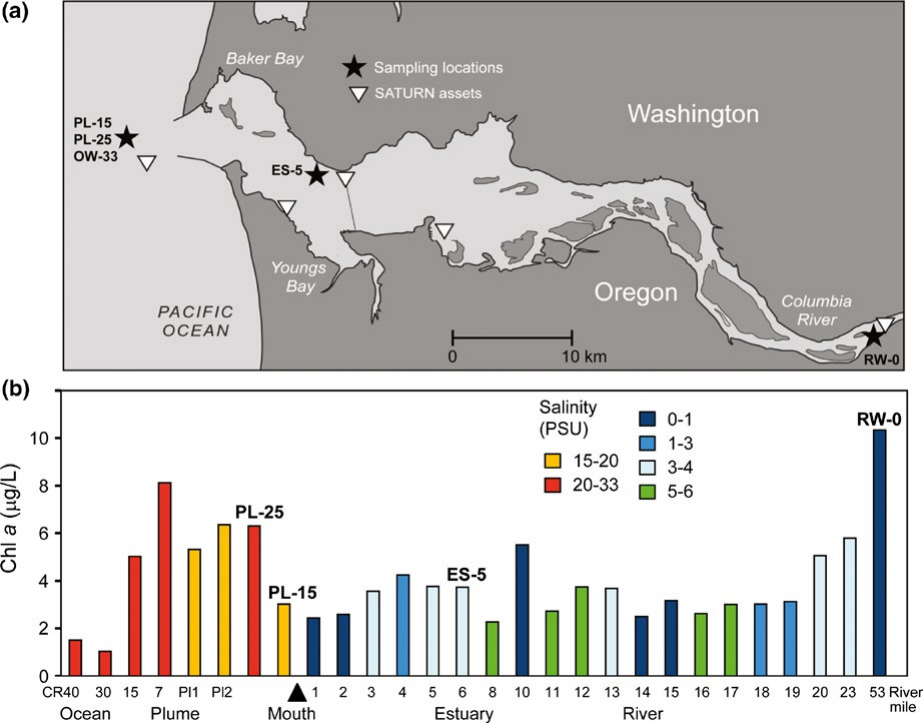
(a) Contour map of the lower Columbia River estuary. The locations of water column sample collections in the river, estuary, and plume are shown with stars. The white triangles show locations of SATURN endurance stations. (b) Chl *a* concentrations (μg/L) in the upper 1 m of water column measured in whole water samples collected along an ocean to river transect conducted between 31 July and 7 August 2010. The samples are colored according to the water salinity values as shown in the inset legend. The *x*‐axis shows locations left to right along the ocean to river transect (not shown to scale for distance). Water samples include collections from the Columbia River Line (CR40, 30, 15, and 7), the river plume (Pl1, Pl2, PL‐25, and PL‐15), and the estuary and river, with river locations marked as river miles (RM) starting from the mouth (shown with the arrowhead) and ending at RM 53 (RW‐0 sample collection location)

Properties of the water associated with sample collections were analyzed using physical and biogeochemical data obtained with the Science and Technology University Research Network (SATURN; Baptista et al., 2015) integrating networked sensors (SATURN monitoring stations, Figure 1a), mobile platforms, research campaigns, and numerical models (Fortunato et al., 2013; Gilbert et al., 2013; Karna et al., 2015; Roegner et al., 2011). Five water samples from the Columbia River (RW‐0, with salinity of 0 PSU), estuary (ES‐5), plume (PL‐15 and PL‐25), and adjacent coastal ocean (OW‐33) were collected between 1 and 8 August 2010, at the locations shown in Figure 1a (Fortunato & Crump, 2015). The samples were size fractionated using 3 and 0.2 μm filters. Because Fortunato and Crump (2015) focused on the free‐living bacteria of the Columbia River estuary, the >3 μm prefilter fractions originally collected from the water samples were unfortunately not retained for subsequent analysis. DNA was isolated from the 0.2 to 3 μm fractions and an aliquot of unfiltered water was analyzed for biogeochemical variables as described previously (Fortunato & Crump, 2015).

### 2.2 Sequencing and metagenome analysis

Sequencing of total DNA was done with Hi‐Seq 1000 (Illumina, San Diego, CA). Metagenomes, assembled as described previously (Fortunato & Crump, 2015; Smith et al., 2015), were analyzed using the Integrated Microbial Genomes with Microbiome Samples–Expert Review web server (IMG/M‐ER of the DOE Joint Genome Institute, https://img.jgi.doe.gov/cgi-bin/mer/main.cgi; Markowitz et al., 2012). During this analysis, gene/peptide prediction was done for each assembled contig, and each corresponding gene (including rDNA and predicted peptide coding sequences) was treated as a separate entry. Each coding DNA sequence (CDS, also referred to as gene) within a scaffold was considered as a single entry (a predicted peptide) even if it was assembled from multiple reads. We used this approach because the majority of scaffolds were short (with the average number of genes 1.2–1.7 per contig), and not particularly abundant, due to high complexity of the estuarine microbiota. Table 1 shows the IMG/M‐ER accession numbers (taxon_oid, taxon object identification) for the metagenomes.

**Table 1:** Sample information, metagenome accession numbers, metagenome statistics, and taxonomic structure

Sample name	OW‐33	PL‐25	PL‐15	ES‐5	FW‐0
Collection date (2010)	7 August	7 August	8 August	1 August	4 August
IMG/M‐ER taxon_oid	3300000930	3300000928	3300000929	3300000883	3300000882
Total annotated CDS	729,300	644,095	653,119	520,559	484,692
Total rDNA genes	5,140	5,338	5,567	3,147	3,569
Mean gene size (bp)	466	466	425	416	382
Effective bacterial genome size (Mb)	2.35	2.32	2.13	2.73	2.05
Number of effective genomes	131.12	118.02	119.58	71.67	85.9
Bacteria (%)	90.80	91.12	91.86	90.22	95.04
Archaea (%)	4.45	2.05	2.10	0.43	0.51
Bacillariophyta (%)	0.08	0.07	0.04	3.91	0.04
Other Eukaryota (%)	2.63	2.90	2.20	5.21	2.70
Bacteriophages (%)	1.95	3.76	3.73	0.17	1.62

Metagenomes of the 0.2–3 μm fractions of water column samples: OW‐33, ocean water; PL‐25 and PL‐15, plume; ES‐5, estuarine; RW‐0, freshwater (the exact locations are shown in Figure 1). Gene/CDS annotations were based on ≥30% identity over ≥70% of the alignment length. The taxonomic abundances are shown as percentages of the total number of annotated CDS in each metagenome.

Taxonomic analyses were first done at the domain level using a cutoff of ≥30% predicted amino acid sequence identity over ≥70% of the length of a pairwise alignment for a given gene/CDS with the corresponding top hit reference sequence. The additional cutoffs of ≥60% and ≥90 identity (over ≥70% of the amino acid alignment length) provided family to genus, and species/strain level identifications, respectively (Konstantinidis & Tiedje, 2005). The relative abundance of taxa was calculated in metagenomes as the sum of all corresponding genes divided by the sum total of all annotated CDS (Table 1). Functional comparison of metagenomes was done using normalized difference, *D*‐score and *D*‐rank, calculated as described previously (Markowitz et al., 2008; available in IMG/M‐ER). Effective bacterial genome size (EGS) was calculated using a set of 35 core bacterial marker genes (Raes, Korbel, Lercher, von Mering, & Bork, 2007; Smith et al., 2013; Table 1). The relative abundance values for functional gene categories (COGs, Pfams, or enzymes) were calculated as the total number of predicted peptides or CDS for a category in a given metagenome, divided by the corresponding EGS (Table 1; Smith et al., 2013).

## 3 Results and Discussion

### 3.1 Biogeochemical data on riverine and marine phytoplankton blooms

**Figure 2:**
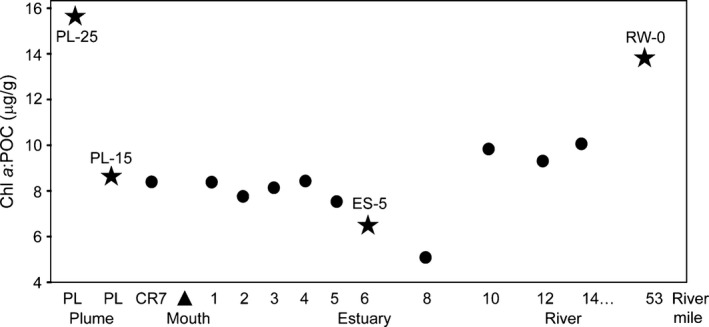
Chl *a*:POC ratios calculated for whole water samples collected along an ocean to river transect in the time period from 31 July to 7 August 2010. Stars show the samples that were subsequently fractionated and used for metagenome sequencing. Circles show the samples that were used only for biogeochemical measurements. The *x*‐axis shows sampling locations along the ocean to river transect (not shown to scale for distance). Water samples collected beyond the mouth in the river plume are indicated with “PL” and “CR” (PL‐25, PL‐15, and CR7), while with locations of samples collected within the estuary and river are marked as river miles (RM) starting from the mouth (shown with the arrowhead) and ending at RM 53 (location of RW‐0 sample collection)

Typical patterns of wind‐driven upwelling were observed along the Oregon coast in the summer of 2010. Nevertheless, real‐time measurements of chlorophyll *a* (chl *a*) concentrations at SATURN observation stations indicated that the annual development of large‐scale oceanic phytoplankton blooms was significantly delayed (compared to a 10‐year average) until after our water sampling campaign was completed at the beginning of August (data not shown). In contrast, riverine phytoplankton bloom development followed the typical annual pattern (Frey et al., 1984; Maier, 2014), with several relatively small blooms observed throughout the summer (data not shown). Laboratory measurements of chl *a* concentrations in 28 discrete whole surface‐water samples collected along a transect from the coastal ocean to the Columbia River at the end of July and beginning of August 2010 (Figure 1b) showed similar amounts in both freshwater and marine end‐members (10 and 6–8 μg L^−1^, respectively). Both end‐members also showed relatively high ratios of chl *a* to particulate organic carbon (chl *a*:POC; 14–16 μg g^−1^). These ratios were calculated for whole water samples (Figure 2) and were well within the range of healthy phytoplankton (estimated as 10–30 μg g^−1^ by (Prahl et al., 1997). This indicates that live phytoplankton were present in both the river and coastal ocean at the time of sampling.

Water samples collected at midsalinity in the plume and estuary, however, had lower chl *a*:POC ratios, indicating the presence of dead and degraded phytoplankton (Prahl et al., 1997). The two samples with the lowest calculated chl *a*:POC ratios included one of the sequenced samples ES‐5 (6.6 μg g^−1^, Figure 2). This low ratio could not be attributed to an increase of POC in the estuary, since POC was 25% higher in the river (748 vs. 567 μg/L; Fortunato & Crump, 2015). Both POC and chl *a* were measured using whole water, revealing that the decreased ratio was associated with a decrease in the content of chl *a* (Fortunato & Crump, 2015).

### 3.2 DNA sequence data on eukaryotic plankton distribution along river to ocean gradient

The estuarine water metagenomes were generated from a relatively small filter size fraction (0.2–3.0 μm) targeting collection of free‐living bacteria. Nevertheless, our previous metagenome data (Smith et al., 2013) and observations by others (Williams et al., 2013) indicated that smaller filter size fractions can also contain DNA from large and/or multicellular organisms, due to leakage of cellular debris and organelles from dead cells. Some eukaryotic organisms may be broken apart by the filtration process during sample collection, or may die in the environment before collection. In the latter case, DNA is attacked by intracellular nucleases upon cell death, and the degradation products leak into the environment (Nielsen, Johnsen, Bensasson, & Daffonchio, 2007), where they serve as an important source of organic matter and are rapidly consumed by bacteria (Dell'anno & Corinaldesi, 2004). In studies examining Bayboro Harbor and Tampa Bay estuarine waters in Florida, DNA turnover time in environmental water was found to be as short as 6.5 hr (Paul, Jeffrey, & Deflaun, 1987). This finding suggests that detection of DNA from large organisms in a small filter size fraction implies relatively recent cellular death. Thus, in addition to the chl *a*:POC ratio, we also evaluated the metagenomes for the presence of phytoplankton genes in the 0.2–3 μm size fractions, to assess their physiological state in the water at the time of sampling.

**Figure 3:**
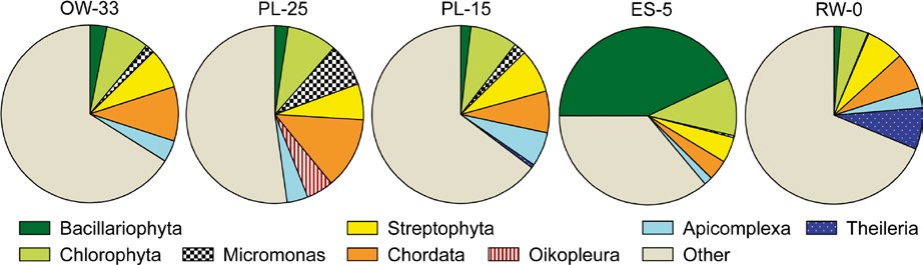
Taxonomic composition of the domain Eukaryota based on predicted gene annotations in assembled metagenomes. Annotations were based on ≥30% identity over ≥70% of the alignment length, with three exceptions: genera *Micromonas*,* Oikopleura*, and *Theileria*. For these genera, predicted peptides were annotated at ≥90% identity (over ≥70% of the alignment length), and their numbers were subtracted from the numbers for the corresponding phyla (Chlorophyta, Chordata, and Apicomplexa for *Micromonas*,* Oikopleura*, and *Theileria*, respectively). The metagenome names provide information on sampling locations and water salinity (PSU; OW‐33, ocean water; PL‐25 and PL‐15, plume; ES‐5, estuarine; RW‐0, freshwater)

All five water metagenomes, including riverine RW‐0 (0 PSU), estuarine ES‐5 (5 PSU), plume PL‐15 (15 PSU), PL‐25 (25 PSU), and oceanic OW‐33 (33 PSU) samples (Figure 1a, Table 1), contained diverse genes predicted to be eukaryotic phyla (Figure 3). Predicted genes corresponding to freshwater eukaryotic plankton were most abundant in the riverine RW‐0 metagenome, while sequences corresponding to typical marine eukaryotic plankton were found only in the plume and coastal ocean, but not in freshwater samples (Micromonas and Oikopleura in Figure 3). In general, the number of sequences representing eukaryotes was relatively low (in the range of 2%–3% of total annotated CDS) compared to bacterial sequences, which constituted >90% of total annotated CDS (Table 1). An exception to this was the ES‐5 metagenome, which contained >9% of sequences annotated as Eukaryota, with almost 4% of those belonging to Bacillariophyta (diatoms, Table 1 and Figure 3). This uneven distribution was not observed for phyla that were anticipated to have broad distributions. For example, Streptophyta (identified as terrestrial by taxonomic annotations), likely originating from both vegetative debris and wind‐ and water‐dispersed pollen and spores (Heusser & Balsam, 1977; Smith et al., 2013), were detected at similar abundances in all samples (Figure 3).

### 3.3 Diatom dynamics in the Columbia River estuary

Genes annotated as belonging to phytoplankton were almost entirely absent in four of the five metagenomes, even though the corresponding whole water samples had relatively high chl *a* values (Figure 1b). This indicated that prefiltration with 3‐μm pore‐size filters successfully removed living phytoplankton cells. In contrast, the ES‐5 metagenome contained large numbers of predicted Bacillariophyta genes (20,370 in total, 43% of eukaryotic [Figure 3] and 4% of all annotated CDS [Table 1]), despite having 2–3X less chl *a* relative to RW‐0 and PL‐25 samples (Figure 1b). The leakage of diatom genes into the ES‐5 small size fraction could not be due to mechanical disruption from the filtration process, because it did not occur in other samples filtered the same way. This result was instead consistent with the lysis of riverine diatoms in low‐salinity waters of the lower estuary as observed previously (Lara‐Lara et al., 1990; Prahl et al., 1997), and subsequent passage of cellular debris, organelles containing DNA, and/or the DNA itself through the prefilter. Diatom cell lysis in the ES‐5 whole water sample is also consistent with the observed decrease in chl *a* concentration relative to the riverine and oceanic end‐member samples (Figure 1b).

Molecular analysis of *rbcL* genes encoding the large subunit of ribulose 1,5‐bisphosphate carboxylase (RuBisCO) was useful for identifying the diatom taxa present in ES‐5 (Evans, Wortley, Simpson, Chepurnov, & Mann, 2008). The two longest predicted *rbcL* peptides (91 and 153 amino acids) corresponded to *Aulacoseira granulata* and *Asterionellopsis glacialis* (with 100% and 99% sequence identities observed, respectively, over the full length of the fragments). Both of these species are typically abundant in the Columbia River estuary, mouth, and plume (Breckenridge et al., 2014; Maier, 2014). During summers, freshwater *Aulacoseira* spp. blooms develop in the river and are transported to the estuary, where they are the dominate phytoplankton species observed and are often found together there with marine *A. glacialis* from the coastal ocean (Breckenridge et al., 2014). Several other known chloroplast‐encoded genes in addition to *rbcL* were also identified in the ES‐5 metagenome, including eight predicted to encode photosystem I PsaA and PsaB proteins (data not shown). Taken together, these data suggest that the presence of diatom DNA did not originate from living diatom cells, but rather from leaked chloroplasts of dead phytoplankton.

Generally, both marine and freshwater diatom taxa are considered ill adapted to intermediate water salinities and perish in the estuary (Frey et al., 1984; Haertel et al., 1969; Herfort, et al., 2011). Once released from dead cells, the 0.5–2 μm chloroplasts can persist in the environment (due to protection afforded by their four membranes; Bedoshvili, Popkova, & Likhoshway, 2009), and subsequently pass through 3‐μm prefilters collecting on 0.2 μm filters. In prior work, we observed a much higher abundance of diatom chloroplast DNA relative to nuclear DNA in the estuarine water column (Crump, Hopkinson, Sogin, & Hobbie, 2004) and in unfractionated sediment (Smith et al., 2015).

### 3.4 Relative abundance of bloom‐associated bacteria

**Figure 4:**
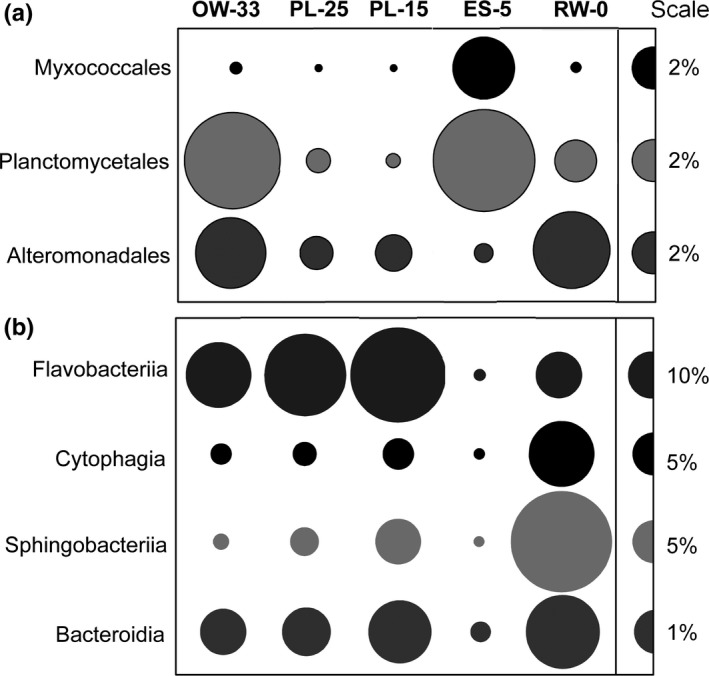
Relative abundance of bacterial taxa based on predicted gene annotations in assembled metagenomes: (a) bacterial orders predicted to be involved in phytoplankton turnover; and (b) four classes of the phylum Bacteroidetes. Abundance values are expressed as percentages of the corresponding genes out of the total annotated CDS in a given metagenome (with annotations based on ≥30% identity over ≥70% of the alignment length). The abundance values are proportionate to the bubble width. The scale for each row is indicated with a half‐bubble to the right, and its diameter corresponds to a percentage value shown with a number. The metagenome names provide information on sampling locations and water salinity (PSU; OW‐33, ocean water; PL‐25 and PL‐15, plume; ES‐5, estuarine; RW‐0, freshwater)

Most of the heterotrophic bacterial groups typically associated with phytoplankton blooms in oceanic and estuarine ecosystems (Amin, Parker, & Armbrust, 2012; Buchan et al., 2014; Pizzetti et al., 2011; Smith et al., 2015; Williams et al., 2012, 2013) were abundant in our dataset (Figure 4). Sequences representing these taxa, however, were distributed differentially across the metagenomes (Figure 4a). Except for lower numbers in freshwater (approximately 1% of total bacterial CDS), metagenome sequences representing the family Rhodobacteraceae were more or less evenly distributed in abundance along the salinity gradient (Fortunato & Crump, 2015).Genes classified to the order Alteromonadales were most abundant in the end‐member metagenomes, RW‐0 and OW‐33 (3.4% and 3.7% of total bacterial CDS, respectively, vs. 1.5% in PL‐15 and PL‐25), and were reduced in the ES‐5 sample (0.9%; Figure 4a). Genes classified to the phylum Planctomycetes were overrepresented in the ES‐5 (1%–2%) and OW‐33 (~5%) metagenomes (Figure 4a). Previously, environmental Planctomycetes taxa were shown to reside on surfaces of algal particles (Lage & Bondoso, 2011), thus their enrichment in the metagenomes may be associated with the presence of phytoplankton debris in the small fractions of these samples.

The phylum Bacteroidetes contained the most abundant sequences of bloom‐associated bacteria, representing 20%–32% of all annotated CDS in four of the five metagenomes (Figure 4b). This taxon is abundant in estuaries around the world (Kirchman, 2002). In earlier studies on the Columbia River and in Delaware Bay, Bacteroidetes taxa showed little (Fortunato et al., 2013) to no (Campbell & Kirchman, 2013) difference, respectively, in abundance along estuarine salinity gradients. Thus, it was surprising to discover that genes from Bacteroidetes taxa were only 1/5 to 1/3 as abundant in ES‐5 as they were in the four other metagenomes (Figure 4b). This dramatic decrease was observed for genes corresponding to all four classes of the phylum Bacteroidetes (Figure 4b).

**Figure 5:**
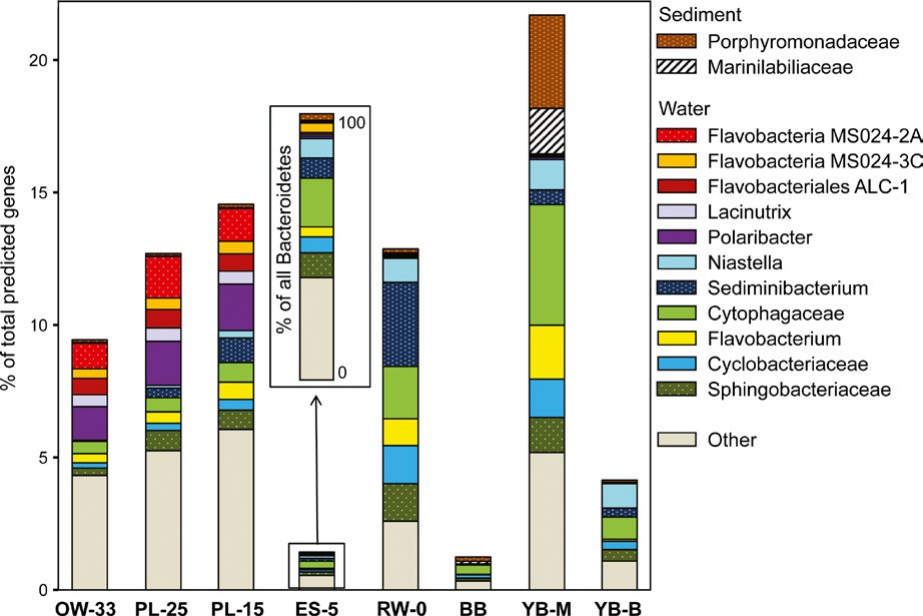
Taxonomic composition of the Bacteroidetes families and genera based on predicted gene annotations in assembled metagenomes. Predicted peptides were annotated using the cutoff of ≥60% identity over ≥70% of the alignment length. Relative taxon abundance is expressed as a percentage of the corresponding genes over the total annotated CDS in a metagenome. The metagenome names provide information on sampling locations and water salinity (PSU; OW‐33, ocean water; PL‐25 and PL‐15, plume; ES‐5, estuarine; RW‐0, freshwater), while the names for metagenomes of lateral bay sediments are those given in Smith et al. (2015) to describe locations (BB for Baker Bay, YB‐M for Youngs Bay mouth, and YB‐B for Youngs Bay back). The inset rectangle shows an enlarged view of Bacteroidetes taxon abundances (expressed as the percentages of all Bacteroidetes genes) in the ES‐5 metagenome. Two taxa were selected as specific for the lateral bay sediments based on their high abundance in the YB‐M metagenome

It is noteworthy that although abundance was much lower in this ES‐5 metagenome, the taxonomic composition of Bacteroidetes (the horizontal bar inset in Figure 5) was very similar to that of the freshwater (RW‐0) sample. This finding suggested relatively uniform depletion occurred across taxa. These data can be explained by the depletion of free‐living stocks of Bacteroidetes due to their attachment to diatom cellular debris (from recently lysed diatoms) in low‐salinity waters. An attached lifestyle has been observed for many diverse Bacteroidetes taxa that typically prefer to grow on algal surfaces and in the phycosphere of senescent phytoplankton (Amin et al., 2012; Bennke, Neu, Fuchs, & Amann, 2013; Buchan et al., 2014; Fernandez‐Gomez et al., 2013; Longford et al., 2007; Nedashkovskaya et al., 2004; Williams et al., 2012, 2013). During bloom utilization in the water column, various Bacteroidetes taxa are known to colonize POM and switch from free‐living to particle‐attached life style (Gonzalez et al., 2008, 2011; Kirchman, 2002). Our previous metagenome data (Smith et al., 2013) also showed pronounced enrichment of typically phytoplankton‐associated Flavobacteria taxa (Bacteroidetes phylum) in the large particulate (3–200 μm) fraction of estuarine water samples containing a degrading bloom. Additional (non‐Bacteroidetes) taxa are also known to switch to a particle‐attached lifestyle with phytoplankton availability (Morris, Longnecker, & Giovannoni, 2006; Pizzetti et al., 2011), which may explain the reduced abundance of members of the order Alteromonadales in the ES‐5 metagenome (Figure 4a), although this reduction was less dramatic than for Bacteroidetes (Figure 4b).

### 3.5 Comparison of Bacteroidetes in estuarine water vs. sediment metagenomes

Our previous results from analyzing sediment metagenomes collected in two Columbia River estuary lateral bays showed that phytoplankton bloom deposition observed at the mouth of Youngs Bay coincided with a dramatic increase in Bacteroidetes abundance (to almost 60% of all bacterial peptides; Smith et al., 2015). This increase was observed for a large number of diverse taxa associated with both sediment and freshwater/low‐salinity habitats of the Columbia River estuary (see the sample YM in Figure 5). Comparing the most abundant Bacteroidetes taxa at the family and genus levels (≥60% sequence identity) in these sediment metagenomes (Smith et al., 2015) versus the water column metagenomes showed both habitat‐specific and common abundance patterns (Figure 5). Two families, Porphyromonadaceae and Marinilabiliaceae, were observed almost exclusively in the sediment (Figure 5). Several taxa, including the genus *Polaribacter*, were observed mainly in plume and oceanic water metagenomes (Figure 5). In contrast, many other taxa, including Flavobacterium, Sediminibacterium, Cytophagaceae, and Cyclobacteriaceae were enriched in both low‐salinity water and the YB‐M sediment sample containing a degraded diatom bloom (Figure 5). Although their abundance varied, these taxa showed considerable tolerance to a wide range of water salinities from 0 to 25 PSU (Figure 5). Overlapping enrichment patterns between the water column and sediment metagenomes may indicate that the estuarine POM turnover is mediated by a variety of Bacteroidetes taxa originating from different habitats.

### 3.6 Functional gene comparisons by D‐rank analysis

**Figure 6:**
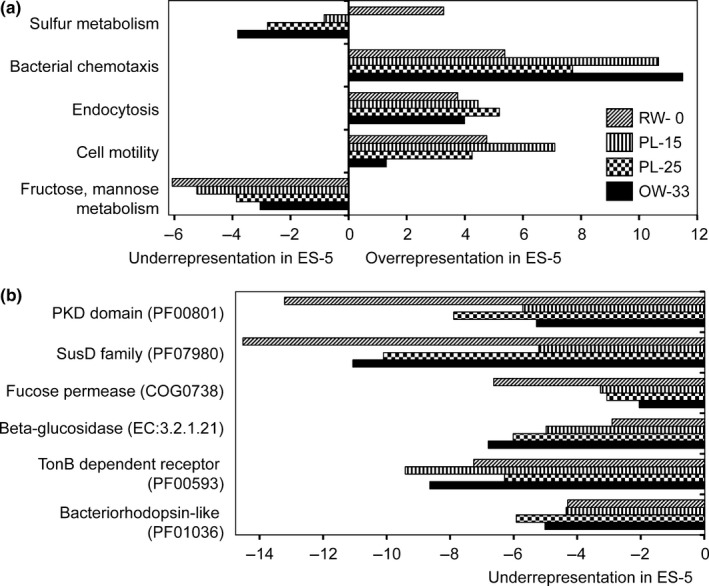
Comparisons of water metagenomes using relative abundance of functional gene categories expressed as *D*‐rank values (bar graphs). For each functional category, positive and negative *D*‐rank values correspond to overrepresentation and underrepresentation, respectively, in the ES‐5 reference when compared to each of the other “test” metagenomes. These values are shown with bars that are coded by the names of test metagenomes. (a) Large functional gene categories were selected using the KEGG pathway classification based on the ENZYME (EC) database. (b) Specific functional gene categories shown from other studies to be enriched in Bacteroidetes genomes were selected from the ENZYME (EC), COG, and PFAM (PF) databases for analysis

We compared the functional gene complement of the ES‐5 metagenome to the four other metagenomes using IMG/M‐ER normalized difference, or *D*‐score and *D*‐rank (Markowitz et al., 2008). A significant *D*‐rank for a functional category (>2.33, with *P*s<0.009) indicated over‐ (positive *D*‐rank) or under‐ (negative *D*‐rank) representation in the “query,” ES‐5, relative to a given “reference” metagenome (Figure 6). Categories showing significant changes in abundance included “sulfur metabolism,” which showed clear association with water salinity. Relative to ES‐5, this functional category was underrepresented in the freshwater (RW‐0) metagenome, while all metagenomes from high‐salinity water showed overrepresentation (Figure 6a). “Bacterial chemotaxis” and “endocytosis” categories were overrepresented in the ES‐5 metagenome (Figure 6a), which is consistent with increased propagation of heterotrophic bacteria attracted to the POM and dissolved organic matter (DOM) from degraded diatoms. Interestingly, the category “fructose and mannose metabolism,” including genes involved in phytoplankton cell wall degradation (Smith et al., 2015), was underrepresented in the ES‐5 metagenome relative to the other water column metagenomes (Figure 6a).

Bacteroidetes genomes contain large suites of genes involved in degradation of high‐molecular‐weight polymers, cell adhesion and attachment, and photoheterotrophy (Fernandez‐Gomez et al., 2013; Gómez‐Pereira et al., 2012; Gonzalez et al., 2011). Many of these genes are found in higher numbers in Bacteroidetes genomes relative to other common environmental bacteria (Fernandez‐Gomez et al., 2013; Gómez‐Pereira et al., 2012; Gonzalez et al., 2011). This list includes genes encoding (1) cell/cell adhesion‐related PKD (polycystic kidney disease) domain proteins; (2) polysaccharide degradation enzymes of the SusD (Sus, starch utilization system) family, and the two CAZymes fucose permease and beta‐glucosidase; (3) TonB‐dependent receptors associated with the Sus membrane degradation system; and (4) proteorhodopsin‐like proteins involved in photoheterotrophy. Analysis of these functional gene categories using the *D*‐rank approach showed that they were grossly underrepresented in ES‐5 relative to the other metagenomes (Figure 6b).

The depletion of genes involved in diatom bloom utilization that are also known to be enriched in Bacteroidetes genomes is consistent with the taxonomic data indicating that the related taxa were depleted in the ES‐5 (Figure 4). Several equally plausible mechanisms may have contributed to Bacteroidetes depletion, including a switch from a free‐living to an attached lifestyle upon phytoplankton cell death resulting in their removal from the 0.2 to 3 μm fraction by the prefilter (as described above), and other scenarios that are described below. Other heterotrophic bacteria may have also been involved in the diatom bloom degradation at different stages of this process, however we did not observe these as major players in the metagenomes, as their sequences were identified at an order of magnitude lower relative abundance compared to Bacteroidetes.

### 3.7 Analysis of viral genes across metagenomes

All metagenomes except for ES‐5 contained many genes annotated as viruses (1.6%–3.8% of all annotated CDS; Table 1), mostly of the order Caudovirales (tailed bacteriophages) at the level of ≥60% identity. Since free bacteriophage particles are too small to have been collected on the 0.2‐μm filter, the phage DNA must have originated from lysogenic viruses collected as part of host bacterioplankton DNA. The apparent correspondence between Bacteroidetes and phage depletion suggests possible pathogen–host interactions for these two taxonomic groups. Phages capable of infecting various Bacteroidetes taxa are widely distributed in marine habitats, and a single Bacteroidetes strain may be coinfected by multiple viral strains (Holmfeldt, Middelboe, Nybroe, & Riemann, 2007; Holmfeldt et al., 2013). Unfortunately, a lack of reference information for most Bacteroidetes‐infecting phages (Holmfeldt et al., 2013) precluded detailed taxonomic annotations of virus genes.

Phages are known to control bacterial community composition and abundance through host‐specific infection and lysis (Fuhrman, 1999; Holmfeldt et al., 2013; Miki & Jacquet, 2008). During phytoplankton blooms in coastal areas, lytic phage production is often positively correlated with high chlorophyll concentrations, with phytoplankton particles serving as hotspots for both bacterial host propagation and phage‐mediated bacterial cell lysis (Mari, Kerros, & Weinbauer, 2007; Riemann & Grossart, 2008; Steward, Wikner, Cochlan, Smith, & Azam, 1992). These processes provide an important mechanism of DOM release through the “viral shunt,” which is part of the microbial loop (Buchan et al., 2014; Fuhrman, 1999; Suttle, 2005). Thus, the Bacteroidetes depletion observed in the ES‐5 metagenomes may have been caused, in part, by lytic phage infection that was induced during bacterial colonization of dead diatom cells. However, direct evidence of lytic infection could not be detected in the ES‐5 metagenome because free phages released upon host lysis would have passed through the 0.2‐μm filter.

### 3.8 Contribution of predation to Bacteroidetes depletion in ES‐5

**Figure 7:**
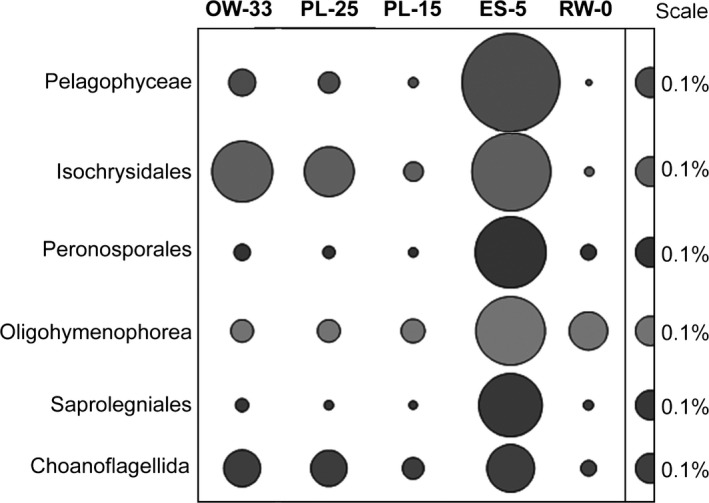
Relative abundance of Protist taxa based on predicted gene annotations in assembled metagenomes. Relative abundance for each family was calculated as the percentage of corresponding genes (with ≥60% sequence identity over >70% of the alignment length) in all annotated CDS in a given metagenome. The abundance values are proportionate to the bubble width. The scale for each row is indicated with a half‐bubble to the right, and its diameter corresponds to a percentage value shown with a number. The metagenome names provide information on sampling locations and water salinity (PSU; OW‐33, ocean water; PL‐25 and PL‐15, plume; ES‐5, estuarine; RW‐0, freshwater)

Unlike broadly distributed terrestrial eukaryotic taxa, but similar to the Bacillariophyta, sequences representing a number of other microeukaryotes were substantially overrepresented (range of 1.5–16X) in the ES‐5 metagenome compared to the other four metagenomes. These included free‐swimming flagellates (Pelagophyceae), ciliates (Oligohymenophorea), and heterotrophic nanoflagellates (2–20 μm; Lee, 2008; Figure 7). Some of these organisms have been observed previously in the Columbia River coastal margin during spring diatom blooms in the river (Kahn, Herfort, Peterson, & Zuber, 2014). Many taxa are microphagous heterotrophs, which graze on smaller organisms, or mixotrophic protists (capable of both phagocytosis and phototrophy), which contribute to the planktonic food web as bacterivorous and herbivorous consumers (Massana, Terrado, Forn, Lovejoy, & Pedrós‐Alió, 2006).

Another taxon involved in grazing and predation, the bacterial order Myxococcales, accounted for 3% of all predicted peptides and was also highly enriched (10X) in ES‐5 compared to the other metagenomes (Figure 4a). In fact, the overrepresentation of “cell motility” genes in ES‐5 (Figure 6a) is likely to be explained, in part, by the myxobacteria using gliding motility to facilitate feeding on insoluble organic particles (Eloe et al., 2011). In aquatic systems, Myxococcales taxa are known as bacterivores (Eloe et al., 2011), and thus their higher relative abundance in the ES‐5 metagenome suggests active predation on the bloom‐associated bacteria.

Furthermore, in a laboratory seawater mesocosm, the sudden and short‐term decrease in Bacteroidetes abundance observed at the onset of a phytoplankton bloom (Riemann, Steward, & Azam, 2000) was also partially attributed to the activity of heterotrophic flagellates, which play an important role in nutrient remineralization (Massana et al., 2006). In pelagic ecosystems, a significant correlation has been observed between bacteriophage and protozoan predator/grazer abundances, both of which contribute significantly to bacterial depletion (Miki & Jacquet, 2008). Thus, rapid growth of heterotrophic bacteria (including Bacteroidetes) on decaying phytoplankton particles likely attracted estuarine predators and grazers, including heterotrophic protists, ciliates, flagellates, and myxobacterial bacterivores, all highly enriched in the ES‐5 metagenome (Figure 4a and 7). The capture of all of these synergistic components contributing to phytoplankton turnover in the dynamic waters of the Columbia River estuary is remarkable.

Taken together, our data suggest a number of mechanisms, including a switch to a particle‐attached lifestyle, grazing/predation, and lytic phage infection, that all may have contributed to Bacteroidetes depletion in the 0.2–3.0 μm fraction of the ES‐5 metagenome by directly reducing the numbers of these bacteria. Additional evidence indicating the overall decrease of heterotrophic bacteria in ES‐5‐associated nonfractionated water samples was obtained using rate measurements of heterotrophic production as described previously (Fortunato & Crump, 2015). The average rates were calculated for two types of estuarine water masses collected within the same 24‐hr period as the ES‐5 sample, low salinity/freshwater (eight samples, 0–3 PSU) and high salinity (two samples, 29–30 PSU). The rates were similar between the two types, 0.75 + 0.19 μg C L^−1 ^hr^−1^ and 0.72 + 0.04 μg C L^−1 ^hr^−1^, respectively. In contrast, the heterotrophic production rate value of 0.48 μg C L^−1 ^hr^−1^ in the ES‐5 water was only about half that of the other water masses. Given this low heterotrophic production in the ES‐5 whole water (which contained the ≥3 μm particles together with any attached bacteria), these data indicate the overall decrease in abundance of heterotrophic bacteria including Bacteroidetes taxa.

### 3.9 Conclusions: a metagenomic window into the estuarine microbial loop

The dynamic nature of the Columbia River estuary and other fast‐flowing estuarine systems presents particular challenges for linking microbial functional processes to specific taxa. Multiple water origins, advection, and mixing all complicate microbiological studies in these estuaries, with very short time windows for capturing the key events influencing microbial processes. Our comparative metagenomic analyses showed that the response of diverse planktonic microorganisms to diatom bloom transport into waters of 3–5 PSU salinity were distinct from those of microorganisms observed in higher salinity waters of the lower estuary. The timing and location of the ES‐5 water collection, in particular, resulted in the effective visualization of many components of the phytoplankton degradative process. Our data suggest several equally plausible scenarios that may follow phytoplankton expiration in low‐salinity waters during the process of bloom utilization by members of the estuarine food web. First, free‐living Bacteroidetes are among the first heterotrophs to colonize the dead phytoplankton particles, and in the process switch from free‐living to a particle‐attached lifestyle; second, the resulting increase in bacterial cell density through rapid growth on the diatom detritus may induce lytic phage infection, with subsequent release of DOM and its entry into the microbial loop (Fenchel, 2008; Fuhrman, 1999), and finally, DOM and POM are transferred to higher trophic levels through the activities of protists and other predators (Azam & Malfatti, 2007; Buchan et al., 2014; Miki & Jacquet, 2008). Each of the outlined scenarios has been previously observed either in laboratory experiments (e.g., particle colonization by Bacteroidetes; Riemann & Grossart, 2008) or in aquatic ecosystems (e.g., virus‐mediated lysis of heterotrophic bacteria, Steward et al., 1992; Mari et al., 2007; protist association with phytoplankton blooms, Kahn et al., 2014). However, to our knowledge, nobody has observed simultaneous occurrence of these processes at the estuarine freshwater–brackish water interface under highly dynamic conditions and constant mixing of riverine and oceanic water masses.

One can argue that additional analysis of the large prefilter fractions of the collected water samples may provide some interesting information on the processes of diatom bloom degradation, however these fractions were not retained during the sample collection. We believe that our study has merit, regardless. Our hypotheses are supported by multiple lines of evidence that were generated from both the metagenomes of the small free‐living sample fractions and biogeochemical measurements of the whole water samples. The proposed scenarios, including particle colonization and retention of these bacteria on the 3 μm prefilters, viral lysis, and grazing/predation, may simultaneously have contributed to the observed depletion of free‐living Bacteroidetes. While it would be useful to measure the relative contributions of each of these plausible scenarios in the course of diatom bloom utilization, this cannot be done by analyzing a few 3‐μm prefilters, given that our previous study (Smith et al., 2013) indicated very high complexity of the large particulate fractions of euphotic water column samples containing diatoms and many other eukaryotic organisms in addition to particle‐attached bacteria and various viruses. In contrast, the small free‐living fractions typically have little or no eukaryotic material (Smith et al., 2013), thus any deviation from this norm is clearly observed and can be interpreted using additional sensor and biogeochemical measurements (from this study) and findings from previous studies.

Research indicates that the Columbia River is a net detritus producer, with the extra detritus originating from lysis of living phytoplankton at or near the halocline and freshwater–brackish water interface (Simenstad et al., 1990). These interfacial/low‐salinity waters are recognized as being highly reactive zones where nonlinear chemical perturbations occur, including the accumulation of phytoplankton detritus from decomposing cells (Fauzi & Mantoura, 1987; Zutic & Legovic, 1987). These interfaces are furthermore stabilized in the salt wedge/highly stratified conditions associated with low river discharge in late summer (Karna & Baptista, 2016) under which our samples were collected. Rapid release of DOM and POM from phyto‐ and bacterioplankton in these zones can increase the retention of elements such as C, N, P, and Fe (Fuhrman, 1999; Weinbauer, Brettar, & Höfle, 2003) and thus may be particularly important in fast‐flowing estuaries with low water retention. Our data together with Fortunato and Crump (2015) provide the first detailed metagenome analysis of bacterial community at or near the halocline in the Columbia River estuary. Previous studies (Prahl et al., 1997) proposed that phytoplankton cells transported into the Columbia River estuary and lysed from osmotic shock become entrained through sedimentation in ETM, resulting in enrichment of bottom water masses with particles of relatively high POC content. Thus, the observed link between bacterial dynamics and decomposition mechanisms at the freshwater–brackish water interface may help to advance our understanding of subsequent organic matter degradation in the ETM and bottom sediments. Our study provides a framework for the development of a high‐throughput sampling scheme examining diatom bloom degradation at the freshwater‐brackish water interface. The metagenome data can be used for future comparisons of bloom utilization between the estuary and plume/coastal ocean at both low and high salinities (5 vs. 15–25 PSU, respectively).

## Conflict of Interest

The authors declare no conflict of interest.
